# Systematic Analyses of the Differentially Expressed Alternative Splicing Events in Gastric Cancer and Its Clinical Significance

**DOI:** 10.3389/fgene.2020.522831

**Published:** 2020-11-17

**Authors:** Changwei Lin, Bowen Yu, Mao Zhang, Yifei Chen, Liang Li, Deze Zhao

**Affiliations:** ^1^School of Life Sciences, Central South University, Changsha, China; ^2^Department of Gastrointestinal Surgery, The Third Xiangya Hospital of Central South University, Changsha, China; ^3^Department of Hepatobiliary and Pancreatic Surgery, Affiliated Hospital of Qingdao University, Qingdao, China; ^4^Department of Otolaryngology-Head Neck Surgery, The Fourth Hospital of Changsha (The Changsha Affiliated Hospital of Hunan Normal University), Hunan Normal University, Changsha, China; ^5^Class 25 Grade 2016, The Five-Year Program in Clinical Medicine, School of Medicine, University of South China, Hengyang, China; ^6^Department of Thoracic Surgery, Xiangya Hospital, Central South University, Changsha, China

**Keywords:** gastric cancer, alternative splicing, differentially expressed AS events, prognostic signature, splicing factor

## Abstract

Accumulation of evidence has indicated a close relationship between alternative splicing (AS) and gastric cancer (GC), whereas systematic analyses of the differentially expressed AS events (DEAS) between GC and normal tissues are lacking. RNA-Seq data and the corresponding clinical information were downloaded from TCGA GC cohort. The percent spliced-in (PSI) value calculated in the GC tissues and normal tissues was employed to quantify the DEAS. Further, survival-associated DEAS and DEAS signatures were identified by univariate and multivariate cox regression analyses. To evaluate the association between DEAS and patients’ clinical features, Kaplan-Meier analysis, receiver operator characteristic (ROC) curve, Cox proportional regression and nomograms incorporating the DEAS signatures were performed. DEAS and their splicing networks were finally analyzed by bioinformatics methods. In addition, we use the method of random grouping to divide the samples into the training group and the test group. The final results of the two groups are consistent. After strict filtering, a total of 44,935 AS events were identified, among which 11,141 DEAS were preliminarily screened from 5032 genes. A total of 454 DEAS was associated with OS, and 872 DEAS were associated with DFS. The final prognostic signatures were constructed from the survival-associated DEAS with an area under the receiver operating characteristic (ROC) curve (AUC) greater than 0.6. Only ES in ABI1 was simultaneously associated with OS and DFS. Finally, we identified the splicing correlation network between the prognostic splicing factors (SF) and DEAS in GC. Our study provided a systematic portrait of survival-associated DEAS in GC and uncovered splicing networks that are valuable in deciphering the underlying mechanisms of AS in GC.

## Introduction

Gastric cancer (GC) is the fifth diagnosed cancer and the third leading cause of cancer-related death worldwide (Bray et al., 2018). Despite advances in screening, diagnosis, and curative resection, the clinical outcomes for individual patients with GC remain unsatisfactory. More seriously, more than 25% of GC patients with resectable tumors will develop recurrence (Wang A. et al., 2016). Thus, further comprehensive understanding of the relationship between the biological mechanisms involved in GC progression and the corresponding clinicopathological characteristics is a vital step to identify novel biomarkers, develop targeted therapy and improve prognosis of GC patients.

Accumulating evidence has revealed that both genetic alterations (Zhang et al., 2018) and epigenetic alterations (Fu, 2015) play key regulatory roles in GC pathogenesis. For example, the molecular subtypes of GC were classified by Kim Y. et al. (2017) through the comprehensive analysis of genetic alterations in Korean GC patients, which provided a critical starting point for the design of more appropriate clinical trials. Additionally, studies involving epigenetic alterations, including methylation, acetylation, histone modifications, etc., have also been widely performed (Patel et al., 2017). The results obtained from the studies mentioned above not only identified GC-related alterations in a number of oncogenes, such as KRAS (Till et al., 2017), c-MET (Bradley et al., 2017), and c-Myc (Khanna et al., 2009), but also revealed the complicated relationship that govern GC malignant progression. These studies, although with promising results, often showed diametrically opposite results. For example, (Clohessy and Pandolfi, 2009) identified p63 as a potent suppressor of metastasis, while (Malaguarnera et al., 2005) showed that p63 may promote thyroid cancer progression. The reason for these kinds of results is that there are many transcripts and variants of these genes (Chen et al., 2018).

Human genome contains over 19,000 protein-coding genes, with over 82,000 transcripts indexed in the current version of GENCODE 27 (Frankish et al., 2019). Alternative splicing (AS), occurring in over 90% of human protein-coding genes, is one of the most extensively applied mechanisms that expands protein diversity in light of the limited number of genes (Yang et al., 2016). During the process of AS, a single RNA precursor can produce structurally and functionally distinct mRNA and protein variants via AS (Revil et al., 2006). Indeed, AS has a profound effect on the biological characteristics of the final protein and accounts for proteome diversity and cellular complexity (Mollet et al., 2010). Although (Shi et al., 2018) and (Lin et al., 2018) conducted transcriptome-wide analysis of the alternative mRNA splicing signature in stomach adenocarcinoma (STAD) tissues from The Cancer Genome Atlas (TCGA), both articles only focused on single AS events. Additionally, systematic analyses of differentially expressed AS events (DEAS) between GC and normal tissues are lacking. Although only protein-coding genes were studied, DEAS could produce differentially expressed transcripts and protein variants.

To further evaluate the potential of DEAS in the prediction of GC prognosis and the regulation of the AS prognostic network by splicing factors (SFs), we systematically analyzed SFs and AS events in GC tissues and normal tissues by analyzing the data provided by the TCGA database. Our results reveal that several GC-related prognostic markers are particularly important in GC, and they may provide clues for therapeutic targets for further validation.

## Materials and Methods

### Data Acquisition and Preprocessing

RNA-Seq data and the corresponding clinical information of the GC patients were downloaded from the TCGA data portal^1^. This study meets the publication guidelines provided by TCGA (Wang Z. et al., 2016). The AS data for each GC patient were analyzed by SpliceSeq. The percent spliced-in (PSI) value calculated by SpliceSeq is used to indicate the reliability of each AS event, and the missing PSI values were imputed using missForest (version 1.4). In this study, (1) a AS event is considered as reliable if more than 75% samples have PSI values. (2) histological diagnosis and complete clinical features. (3) patients with OS less than 30 days and stage IV were excluded. Finally, 304 GC patients were included in analysis cohort. To increase the credibility of bioinformatics analysis, we randomly divide patients into two groups according to a 6:4 ratio (training group vs. test group). The training group was used to perform the relevant analysis, and the test group was applied to verify the conclusion.

### Identification of Differentially Expressed AS Events and Enrichment Analysis

Differentially expressed AS events (DEAS) and Differentially expressed gene (DEG) were analyzed through the limma package (version 3.42.0), and the batch effect was removed by a generalized linear model (Supplementary Figure S1). Adj. *p*.value < 0.05 was used as the threshold to prevent skipping significant changes. The interactive sets between the seven types of reliable DEAS events (Conway et al., 2017) were illustrated by the distinguishable visualization Upset plot (UpSetR, version 1.3.3) and the differences among DEAS and DEG were illustrated using Venn diagrams.

Subsequently, the parent genes of these significantly DEAS were used as the background in enrichment analysis using Metascape (Zhou et al., 2019). Adj. *p*.value < 0.05 was statistically significant.

### Survival Analysis

According to the survival information of two groups, univariate Cox regression and lasso regression (Supplementary Figure S2) were used to determine DEAS, which was significant to overall survival (OS) and disease-free survival (DFS). Further, to establish a rigorous prognostic model, signature DEAS with an area under the receiver operating characteristic (ROC) curve (AUC) greater than 0.6 was selected as candidates for multivariate Cox regression. Then, the risk score for each sample was calculated based on the PSI values of the prognostic DEAS signatures and the corresponding coefficients. GC samples were subsequently divided into two subgroups by the median risk score: high risk group and low risk group. Kaplan-Meier analysis was used to test the model’s ability to distinguish patient’s survival. All the reported *P*-values (if not clearly stated) were less than 0.05. All the analyses were performed using RStudio (version 3.5.2).

### Splicing Factor (SF) Genes and the Potential Regulatory Network

Splicing factor genes were extracted from the SpliceAid 2^2^ database (Giulietti et al., 2013). Based on the RNA-Seq data of tumor and normal tissues, the differentially expressed SF genes (with adj. *p*.value < 0.05) were identified. Then, the correlation between SF genes and signature DEAS events in the prognostic model was analyzed by Hmisc (version 4.3-0). To explore the direct interaction between SF and DEAS, we predicted the binding sites through catRAPID (Livi et al., 2016).

### Development and Apparent Performance of a DEAS-Clinicopathologic Nomogram

We combined multivariable Cox regression analysis and all informative clinicopathologic variables described above to formulate a nomogram for the better prediction of the individualized survival rates of GC patients (Zhang and Kattan, 2017). Backward stepwise variable selection with the Akaike information criterion (AIC) was performed to determine the variables included in the final nomogram (Rousson and Zumbrunn, 2011; Balachandran et al., 2015). Then, the predictive accuracy of the final nomogram was evaluated by Calibration curves. To further identified predictive efficiencies of the model, the Uno’s inverse-probability of censoring weighting estimation of the dynamic time-dependent ROC area under the curve (AUC) values (time span from 0 to 3 years) was calculated with time ROC package (version 0.3) (Blanche et al., 2013).

## Results

### Overview of AS Event Profiling in GC

**FIGURE 1:**
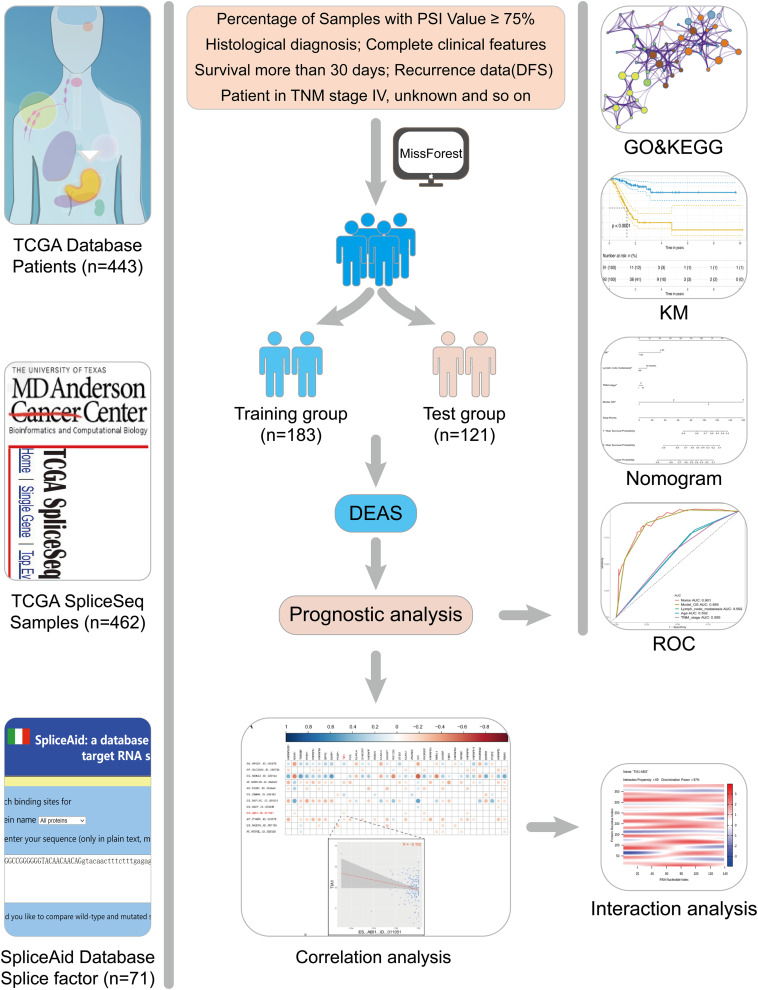
Flowchart of the present study.

**FIGURE 2:**
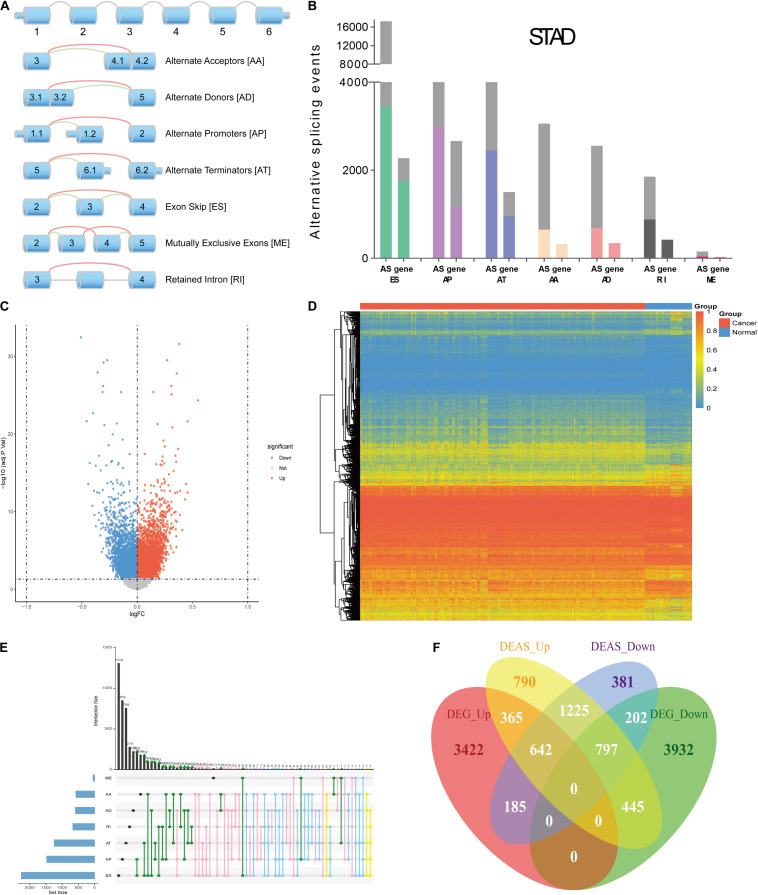
Overview of AS events profiling in GC. **(A)** Illustration of the splicing patterns of seven types of AS events, including alternate acceptor site (AA), alternate donor site (AD), alternate promoter (AP), alternate terminator (AT), exon skip (ES), mutually exclusive exons (ME) and retained intron (RI). **(B)** Seven types of AS events and corresponding genes from the patients in training group are depicted according to a classified *P*-value of 0.05. **(C)** The difference in AS events between GC tissues and normal tissues. The DEAS identified in GC was visualized in a Volcano plot. The red and blue points in the plot represent DEAS with statistical significance (adj *P* value < 0.05). **(D)** Heat map of the DEAS. The horizontal axis shows clustering information of samples divided into two major clusters: GC tissue and normal tissue. The left longitudinal axis showed the DEAS clustering information. The gradual change of color from green to red represents the alteration in DEAS expression from low to high. **(E)** UpSet plot of the interactions between DEAS events and their parent genes. **(F)** Venn diagram demonstrated the intersection set of DEAS and DEG.

The design of this study design is illustrated, as shown in Figure 1. Integrated AS event profiling was curated using AS events data and clinical information obtained from 304 GC patients. The included population comprised 199 male (65.5%) and 105 female (34.5%) patients. Among these patients, 88 (28.9%) patients developed recurrence and 105 (34.5%) died. The clinical information of GC patients is summarized in Supplementary Table S1. In SpliceSeq (Ryan et al., 2016), the AS events are divided into seven types based on the splicing patterns, as illustrated in Figure 2A. By using SpliceSeq on the RNA-Seq data of GC patients, we found a total of 44,935 AS events in 10,256 genes, including 17,274 exon skip (ES) in 2,273 genes, 198 mutually exclusive exons (ME) in 31 genes, 2,738 retained intron (RI) in 658 genes, 9,573 alternate promoter (AP) in 3,844 genes, 8,198 alternate terminator (AT) in 2,468 genes, 3,241 alternate donor site (AD) in 401 genes, and 3,713 alternate acceptor site (AA) in 581 genes, as shown in Figure 2B. The detail of the detected AS events is listed in Supplementary Table S2. Consistent with the previous studies, according to the detected AS events of GC patients, we can also find each gene contains almost four types of AS events, which indicated the gene expression diversity through the different combinations of splicing types. Similarly, the proportion of ES in the detected AS events (nearly 40%) is still the highest among other types of AS events.

Compared with the detected different AS events between primary GC and normal tissues, we identified 11,141 DEAS, which were corresponding to 5,032 genes (Supplementary Table S3), and the GC-specific splice genes ranked according to upregulation and downregulation were labeled in the volcano plots (Figure 2C). Moreover, we used unsupervised hierarchical clustering based on these DEAS to identify that these DEAS were credible (Figure 2D). To further visualize the intersecting sets of each AS type, an Upset plot was generated. As shown in Figure 2E, 27.9% (1405/5032) of the genes contain two or more DEAS and one gene might have up to five types of DEAS. Besides, we also investigate DEAS that occurred in the differently expressed genes (DEG), as shown in Figure 2F, and the details are provided in Supplementary Table S4. The Venn diagrams suggested that when a gene is up-regulated or down-regulated, it’s AS events may change in the same direction or in the opposite direction, which provides an important supplement to clarify the functional changes caused by gene changes.

### Enrichment and Interaction Analysis of DEAS

**FIGURE 3:**
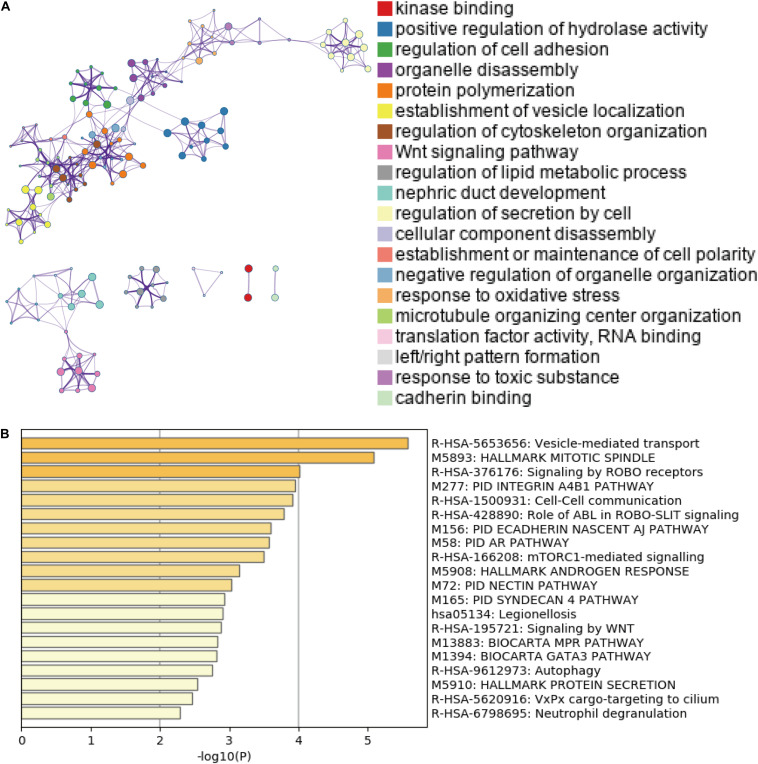
Gene Ontology (GO) and pathway analysis. **(A)** GO annotation of DEAS covering kinase binding, regulation of hydrolase, and regulation of cell adhesion, etc. **(B)** KEGG pathway enrichment analysis of DEAS with enrichment scores.

Accumulating evidence has suggested that AS could directly affect protein expression and function. Therefore, we further examined the potential influence of DEAS by analyzing their corresponding proteins. Figure 3A shows that specific GO categories are enriched in these genes with DEAS, including regulation of cell adhesion, regulation of cytoskeleton organization, response to oxidative stress, etc. Additionally, some pathways were enriched in these genes with DEAS, including pathways in cell-cell communication, mTORC1-mediated signaling, and Autophagy (Figure 3B). Detailed information on GO category and KEGG pathway enrichment are provided in Supplementary Table S5. Both the GO category enrichment results and KEGG pathway enrichment results indicated that the parent genes of DEAS have very close relationship to GC progression.

### The Prognostic Value of DEAS in GC

**FIGURE 4:**
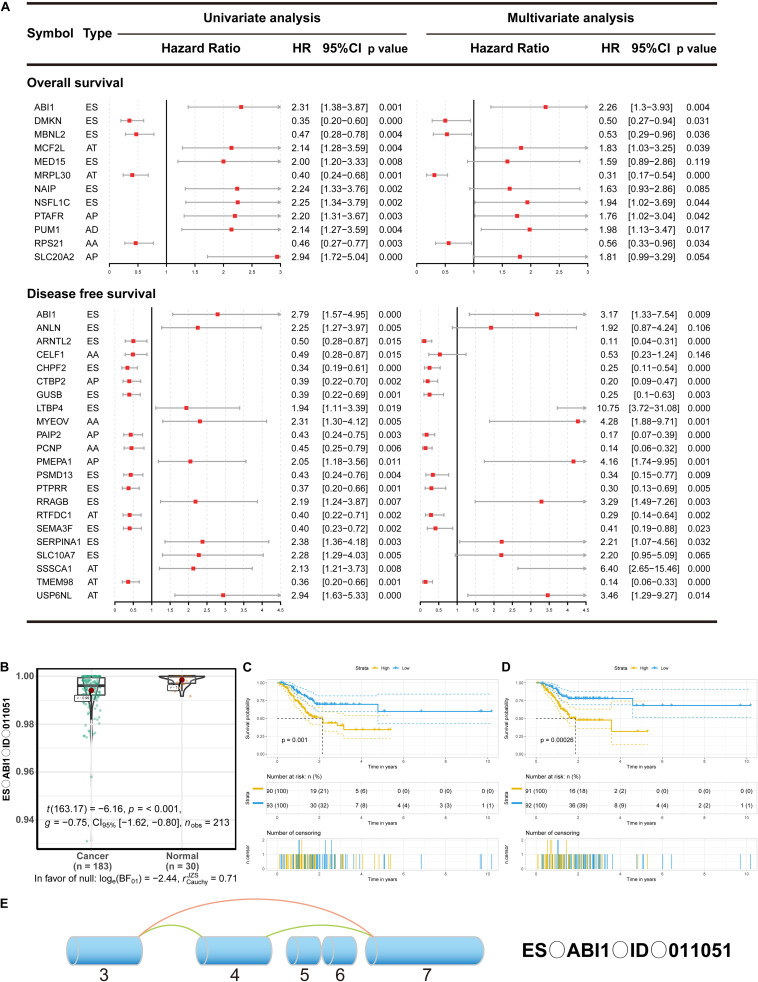
The prognostic value of DEAS in GC. **(A)** DEAS that were simultaneously associated with OS and DFS. Univariate and multivariate analysis of DEAS on OS and DFS. Unadjusted HRs (boxes) and 95% confidence intervals (horizontal lines) limited to DEAS with *P* < 0.05. Box size is inversely proportional to the width of the confidence interval. **(B)** The different PSI values of the ES event in ABI1 in the normal tissues and GC tissues. **(C,D)** Prognostic signatures based on ES events in ABI1 in GC for both OS and DFS. Patients were divided into high- and low-risk groups according to prognostic signatures. The figure contains three parts: [1] survival differences estimated by Kaplan-Meier survival curve; [2] number of patients in different groups; [3] number censored at different times; **(E)** Splice graph of ES events in ABI1. Exons are drawn to scale, and the connecting arcs represent splice paths.

Growing evidence has suggested that cancer-specific splice variants could serve as prognostic biomarkers and therapeutic targets (Robinson et al., 2019). To investigate the relationship between DEAS and prognosis of GC, the univariate survival tests were performed on the clinicopathological features and outcomes in the TCGA GC cohort. As showed in Supplementary Table S6, it can be observe that age (HR = 1.94, 95% CI: 1.05–3.58; *p* = 0.034), lymph node metastasis (HR = 1.84, 95% CI: 1.01–3.44; *p* = 0.046), and TNM stage (HR = 1.47, 95% CI:1.02–2.13; *p* = 0.039) were significantly associated with OS. Meanwhile, lymph node metastasis (HR = 1.31, 95% CI: 1.03–1.66; *p* = 0.028), and sex (HR = 0.42, 95% CI: 0.22–0.82; *p* = 0.011) were significantly associated with DFS. These results indicate that the survival data from the TCGA GC cohort were informative and appropriate for use in further survival analysis. Further, the univariate survival analyses were conducted for OS and DFS. A total of 454 DEAS was significantly associated with OS, and 872 DEAS was significantly associated with DFS. Among these prognosis-related DEAS, there are 61 DEAS that are simultaneously associated with OS and DFS (Supplementary Figure S3). Then the multivariate analysis was performed to identify independent prognostic indicators in GC. As shown in Figure 4A, there were 12 DEAS recognized as independent prognostic indicators for OS and 22 DEAS recognized as independent prognostic indicators for DFS. Among these prognosis-related DEAS, we unexpectedly found that ES in ABI1 was an independent prognostic indicator for both OS and DFS in the GC cohort. Furthermore, to intuitively show the difference in the ES events in ABI1 between GC tissues and normal tissues, we generated scatter plots (Figure 4B). As presented in Figures 4C,D, the DEAS signatures were constructed with an ES event in ABI1. The Kaplan–Meier survival analysis results showed that the ES event in ABI1 was an independent prognostic indicator for both OS (*p* = 0.001) and DFS (*p* = 0.00026) in the GC cohort. Figure 4E depicts ES events in ABI1 in a splice graph.

**FIGURE 5:**
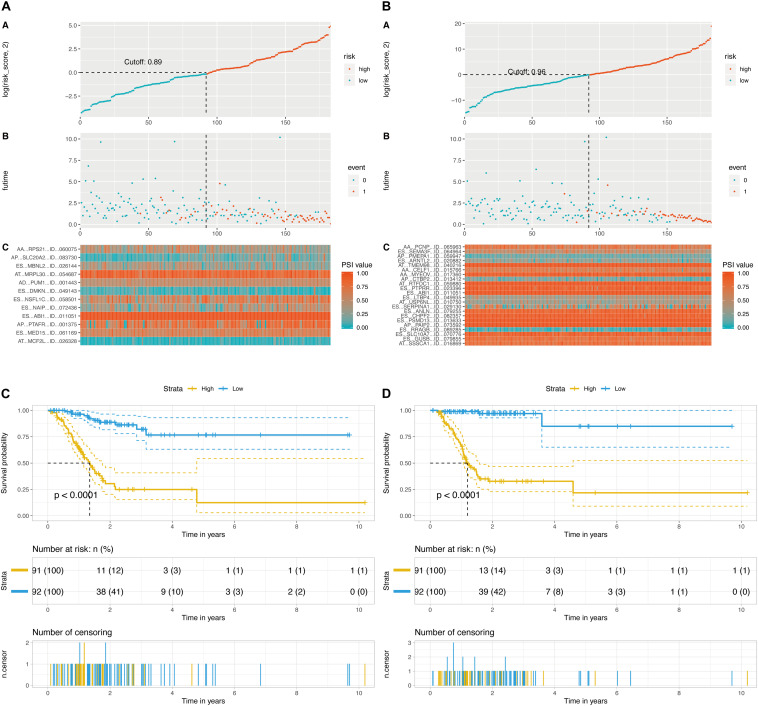
Determination and analysis of the final prognostic models. **(A)** OS-related prognostic model. **(B)** DFS-related prognostic model. High- and low-risk groups were divided based on the median value of risk score. The upper plot illustrated assignment of patients’ survival status and survival times, the middle plot showed the risk score curve, and the bottom heatmap represented splicing distribution of the AS in compound prognostic models. Color transition from blue to red indicates the increasing PSI value of corresponding AS event from low to high. **(C)** KM plot of OS-related prognostic model. **(D)** KM plot of DFS-related prognostic model. The figure contains three parts: [1] survival differences estimated by Kaplan-Meier survival curve; [2] number of patients in different groups; and [3] number censored at different times.

Then, a prognostic model consisting of signature DEAS were identified from the significant DEAS by multivariate Cox regression. Then, the risk score for each sample was calculated based on the PSI values of the prognostic DEAS signatures and the corresponding coefficients. The GC patients were classified into high- and low-risk subgroups based on the median value of risk scores. Figures 5A,B demonstrate that the model could distinguish the survival of patients in the two groups. A Kaplan–Meier survival analysis was employed to assess the relationship between the signatures and prognosis (Figures 5C,D).

### DEAS Correlation Network of Splicing Factors

**FIGURE 6:**
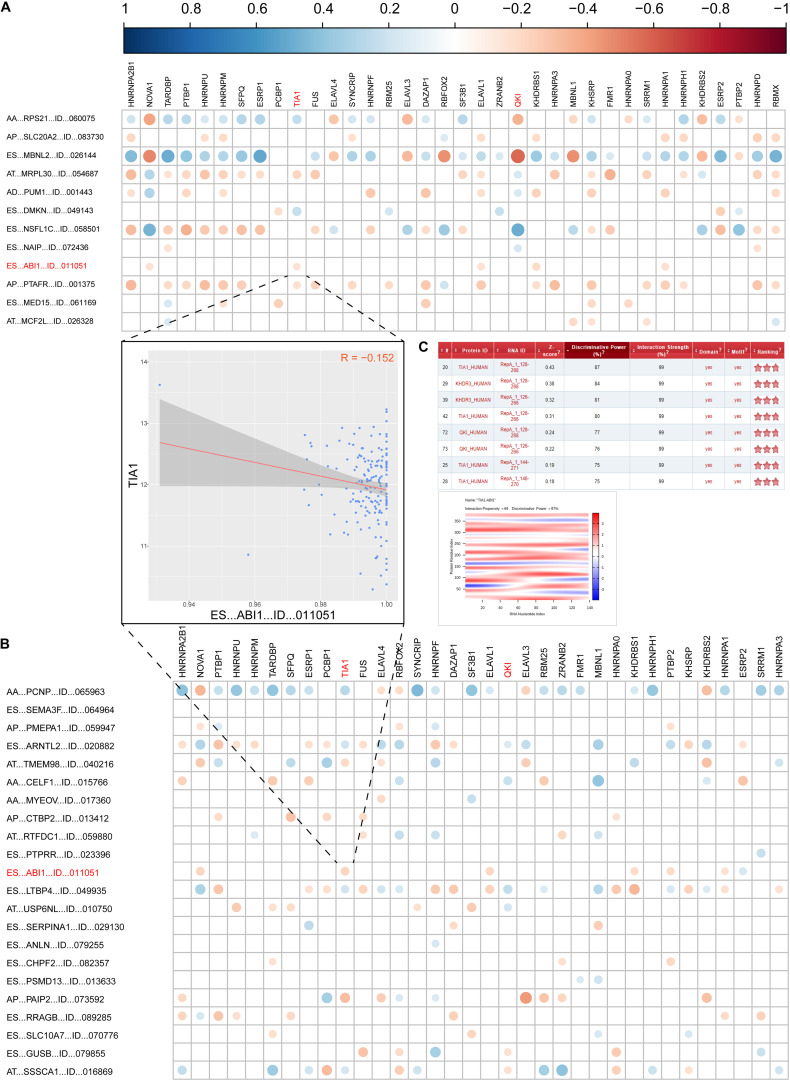
Correlation analysis between splicing factors and DEAS prognostic predictors. **(A)** the correlation between OS-related DEAS and splicing factors. **(B)** The correlation between DFS-related DEAS and splicing factors. The weight of the correlation coefficient was represented by the size and color of the circle. **(C)** The correlation between TIA1 and ES_ABI1. The left graph shows the correlation between the TIA1 and ES_ABI1. The right figure shows the interaction score and site prediction.

As we known, AS events are mainly regulated by splicing factors (SFs). Therefore, we further aimed to find a few key SFs that regulated these DEAS expressions in GC. To resolve this question, we first identified 71 SFs validated in previous study (Supplementary Table S7). Then, the expression of these SFs was determined from the RNA sequencing data in the TCGA GC cohort, and 29 out of the 71 SFs were identified as having differential expression in STAD. The detail of the detected differently expressed splicing factors is listed in Supplementary Table S8. Next, correlation analyses of the expression levels of these 36 SFs and the PSI values of each DEAS were performed in the GC cohort, and a splicing regulatory network was built using the significant correlations (|R|>0.1; *t*-test, *p* < 0.05; Supplementary Table S9). The results imply that 36 SFs are significantly associated with 12 OS-related DEAS and 22 DFS-related DEAS prognostic signatures (*r* > 0.1, *P* < 0.05, Figures 6A,B).

Since ES_ABI1 was related to both the patient’s OS and DFS, we had a keen interest in it. Further, we explored the relationship between ES_ABI1 and SF and found that TIA1 had a direct interaction site with ES_ABI1, and their expression was negatively correlated (Figure 6C). This discovery provides an important foundation for our subsequent experiments.

### Development and Apparent Performance of the DEAS-Clinicopathologic Nomogram

**FIGURE 7:**
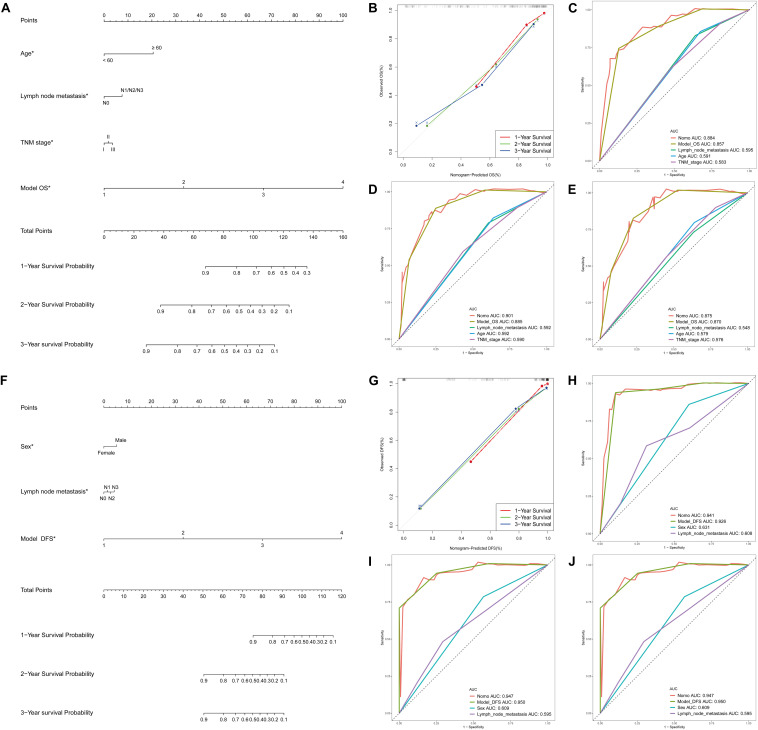
DEAS associated clinicopathologic nomogram for prediction of survival probability in GC patients. **(A)** Development of DEAS associated clinicopathologic nomogram for predicting 1-, 2-, and 3-year OS for GC patients, with age, lymph node metastasis, TNM stage and OS model. **(B)** The calibration curves of 1-, 2-, and 3-year OS nomogram prediction in the GC cohort. The y-axis showed the observed OS, and the red, blue and green line indicated the respective performance of the nomogram with 1-, 2-, and 3-year outcomes in the GC cohort. **(C–E)** ROC curves with calculated AUCs of prognostic signatures built by clinical features, AS prognostic model and the nomogram for risk prediction from 1 to 3 years. **(F)** Development of DEAS associated clinicopathologic nomogram for predicting 1-, 2-, and 3-year DFS for GC patients, with sex, lymph node metastasis, and DFS model. **(G)** The calibration curves of 1-, 2-, and 3-year DFS nomogram prediction in the GC cohort. The *y*-axis showed the observed OS, and the red, blue and green line indicated the respective performance of the nomogram with 1-, 2-, and 3-year outcomes in the GC cohort. **(H–J)** ROC curves with calculated AUCs of prognostic signatures built by clinical features, AS prognostic model and the nomogram for risk prediction from 1 to 3 years.

Supplementary Table S6 showed the results of the univariate Cox analysis of clinicopathologic characteristics. According to the results, age, lymph node metastasis, TNM stage and OS model were independent prognostic factors for OS in the STAD cohort. Similarly, sex, lymph node metastasis and DFS models were independent prognostic factors for DFS. By applying the backward stepwise selection based on optimizing AIC, a total of four variables including age, lymph node metastasis, TNM stage and OS model were finally incorporated in the subsequent OS nomogram construction (Figure 7A), and a total of three variables including sex, lymph node metastasis and DFS model were finally incorporated in the subsequent DFS nomogram construction (Figure 7F). During OS and DFS nomogram, the probability of survival at 1, 2, or 3 years were subject to the calibration cure and indicated a good agreement between the prediction and actual observation (Figures 7B,G). The C-index for OS prediction was 0.832 (95% CI, 0.809–0.855), and the C-index for the DFS prediction was 0.898 (95% CI, 0.879–0.917).

Furthermore, to compare the advantages of clinical indicators and nomograms in evaluating the survival of GC patients, we calculated the ROC curves of clinical features, AS prognostic model and the nomogram in the training group (Figures 7C–E for OS and 7H-J for DFS) and test group (Supplementary Figures S4C,D). The results demonstrated that the AUCs of nomogram in either the training group (AUC > 0.85 from 1 to 3 years) or the test group (AUC > 0.60 from 1 to 3 years) were all obviously greater than those of clinical indicator, which illustrates the robust and valuable predictive efficiency of the nomogram model.

## Discussion

Alternative splicing is the main mechanism that accounts for proteome diversity and cell complexity. Normal tissues can precisely control AS event stability, maintain the usually low spontaneous mutation rate and exert the normal physiological function. However, aberrant AS in cancer tissues is a critical factor in initiation or maintenance of cancer. Preliminary investigations revealed that the potential significance of AS perturbation was involved in the initiation and progression of cancer by generating different mRNA and protein isoforms with diverse functional properties (He et al., 2004; Kameyama et al., 2012). Similarly, several specific AS events in GC have been identified (Lin et al., 2018); we identified 44,935 AS events in GC through the analysis of TCGA data, and ES events were the most frequent AS events. We also found that a single gene might have an average of almost four types of AS events. For example, CD44, an important adhesion molecule serving a critical role in cancer development, has been demonstrated to be associated with risk for several types of cancer via its different splicing modes and contains 3 types splicing modes in GC (AA, AT, and ES). In addition, we observed 47 AS events in CD44 in GC. This result raises the question of whether all these splicing events could play a role in the development of GC. The answer is obvious. Thus, we need a better method to identify significant AS events in GC. Directly comparing gene expression between cancer tissues and normal tissues has been considered an effective approach to screen hub genes involved in the cancer biological processes. Therefore, it is more reliable to identify GC-related AS genes via screening differentially expressed alternative splicing events in GC. Finally, 11,141 DEAS were identified in our study. Interestingly, we found only five DEAS (DYNLL1, SMUG1, PLAGL1, DYNLL1, and SMUG1), which is consistent with the results reported by Shi et al. (2018), who also conducted transcriptome-wide analysis of alternative mRNA splicing signatures in GC (Supplementary Table S10). Therefore, screening DEAS in GC is a different method compared with traditional different expression screening. Consistent with this result, our results showed that only 2636 genes had DEAS simultaneously occurring in DEG.

We then evaluated the potential functions and pathways of the DEAS-associated genes by performing the enrichment analysis. The pathways involving regulation of cell adhesion, which plays a crucial role in tumor progression, invasion and metastasis (Vitte et al., 2005). Furthermore, we also discovered that DEAS-associated genes were positively associated with cell-cell communication, the canonical Wnt signaling pathway, the mTORC1-mediated signaling pathway and autophagy. Notably, immune-related pathways may be involved in GC tumorigenesis.

To further evaluate whether specific DEAS could be used as indicators of GC prognosis, we built prognostic models based on individual AS patterns. Among these DEAS, there are 61 DEAS that are simultaneously associated with OS and DFS according to the univariate analysis. For instance, CAMKK2, overexpressed in 94% (92 of 98) of gastric cancer cases (Subbannayya et al., 2015), contains two variants that differentially modulate neuronal differentiation (Cao et al., 2011). During our study, ES events of CAMKK2 were simultaneously associated with OS (HR = 2.05, 95% CI: 1.23–3.42) and DFS (HR = 1.93, 95% CI: 1.11–3.37).

Interestingly, only ES events in ABI1 were simultaneously associated with OS and DFS according to the univariate survival analyses and multivariate analysis, respectively. ABI1 is a key molecule that coordinates actin cytoskeleton reorganization and growth signaling, which explain the simultaneous dysregulation of PI-3 kinase and actin cytoskeleton in cancer (Kotula, 2012). Cui et al. (2010) found that down-regulation of ABI1 expression plays an important role in tumor progression in gastric carcinoma, and it may be a potential diagnostic biomarker and a promising target for medical treatment. Besides, (Nath et al., 2019) found that ABI1 could suppress EMT of tumor through inhibiting downstream of non-canonical WNT signaling (FYN-STAT3 pathway) and the loss of ABI1 would drive the tumorigenesis of prostate tumors. These studies were consistent with our conclusions. ES_ABI1 was downregulated and significantly related to the patient’s prognosis in our research. In addition, we innovatively found the splicing factor TIA1 which could directly interact with ABI1. This discovery was a supplement to the mechanism and provided a direction for further research.

To predict survival probability, we combined analysis the DEAS signatures and clinical parameters in an inclusive model. Indeed, prognostic nomograms integrated with age, sex, lymph node metastasis and TNM stage were recommended for evaluating individualized survival risk for OS and DFS prediction. Besides, our results also showed a clinical usefulness nomogram in predicting long-term survival probability, especially in 2 and 3 years.

Currently, AS has been considered to be generally regulated by SFs. Therefore, another notable finding of this study was the distinguished splicing correlation network between the expression of SFs and DEAS in GC patients. Correlation analyses between the differential expression levels of SFs and the PSI values of each DEAS were performed in the GC samples, and the results indicated that SFs influence biological process by regulating the AS of many downstream target genes during GC relapse. For example, ES_ABI1 is significantly correlated with the splicing factors NOVA1, TIA1, ELAVL1, KHDRBS1, PTBP2, ESRP2, and HNRNPA1. Among these splicing factors, TIA1 (Yang et al., 2018) and NOVA1 (Kim E. K. et al., 2017) have been proven to promote gastric cancer development via regulating AS. In addition, ELAVL1 (Kim E. K. et al., 2017) and PTBP2 (Cheung et al., 2009) also participate in cancer progression via AS function. The findings of this study shed light on the roles and significance of DEAS in the splicing machinery of GC and can be used to guide the targeting of cancer-specific splicing isoforms as a cancer therapy.

Although our model performs well in GC prognosis prediction, several limitations still need to be improved. First, we need to find other independent cohort of GC patients to prove that the prognostic models proposed here are reproducible. Second, we did not use any number of GC samples to validate the DEAS, so additional studies with larger sample sizes are needed.

In conclusion, to the best of our knowledge, this is the first study to comprehensively evaluate the predictors of long-term survival GC outcomes through molecular analysis of DEAS and to construct an interaction network of DEAS and regulatory SFs. Although the prognostic implications of these potential therapeutic targets for GC still need to be validated by further research, our study still showed that DEAS could serve as diagnostic, predictive and prognostic biomarkers of GC.

## Data Availability Statement

Publicly available datasets were analyzed in this study, these can be found in The Cancer Genome Atlas (https://portal.gdc.cancer.gov/).

## Author Contributions

CL and DZ conceived and directed the project. BY, YC, MZ, and LL designed the study and analyzed the data. CL and BY wrote the manuscript. DZ reviewed the data. All authors have read and approved the final manuscript for publication.

## Conflict of Interest

The authors declare that the research was conducted in the absence of any commercial or financial relationships that could be construed as a potential conflict of interest.

## Footnotes

1 https://portal.gdc.cancer.gov/

2 www.introni.it/spliceaid.html

## Supplementary Material

Supplementary Figure 1 Batches of GC TCGA data.

Supplementary Figure 2 LASSO analysis for DEAS events. **(A,B)** OS-related DEAS. **(C,D)** DFS-related DEAS. In left plot, each curve showed an OS-related DEAS event; 10-fold cross-validation was used to calculate best lambda which leads to minimum mean cross-validated error. The right plot was the partial likelihood deviance of the LASSO coefficient profiles.

Supplementary Figure 3 Forestplot of DEAS that are simultaneously associated with OS and DFS in Univariate COX analysis.

Supplementary Figure 4 | (A,B) KM plot of prognostic model in test group (OS and DFS). The figure contains three parts: [1] survival differences estimated by Kaplan-Meier survival curve; [2] number of patients in different groups; and [3] number censored at different times; **(C,D)** ROC curves with calculated AUCs of prognostic signatures built by clinical features, AS prognostic model and the nomogram for risk prediction from 1 to 3 years (OS and DFS).
