# Supplementary figures and images for: Systematic Analyses of the Differentially Expressed Alternative Splicing Events in Gastric Cancer and Its Clinical Significance
Source: Front Genet. 2020 Nov 17;11:522831. doi: 10.3389/fgene.2020.522831 (PMC7705250; doi:10.3389/fgene.2020.522831)

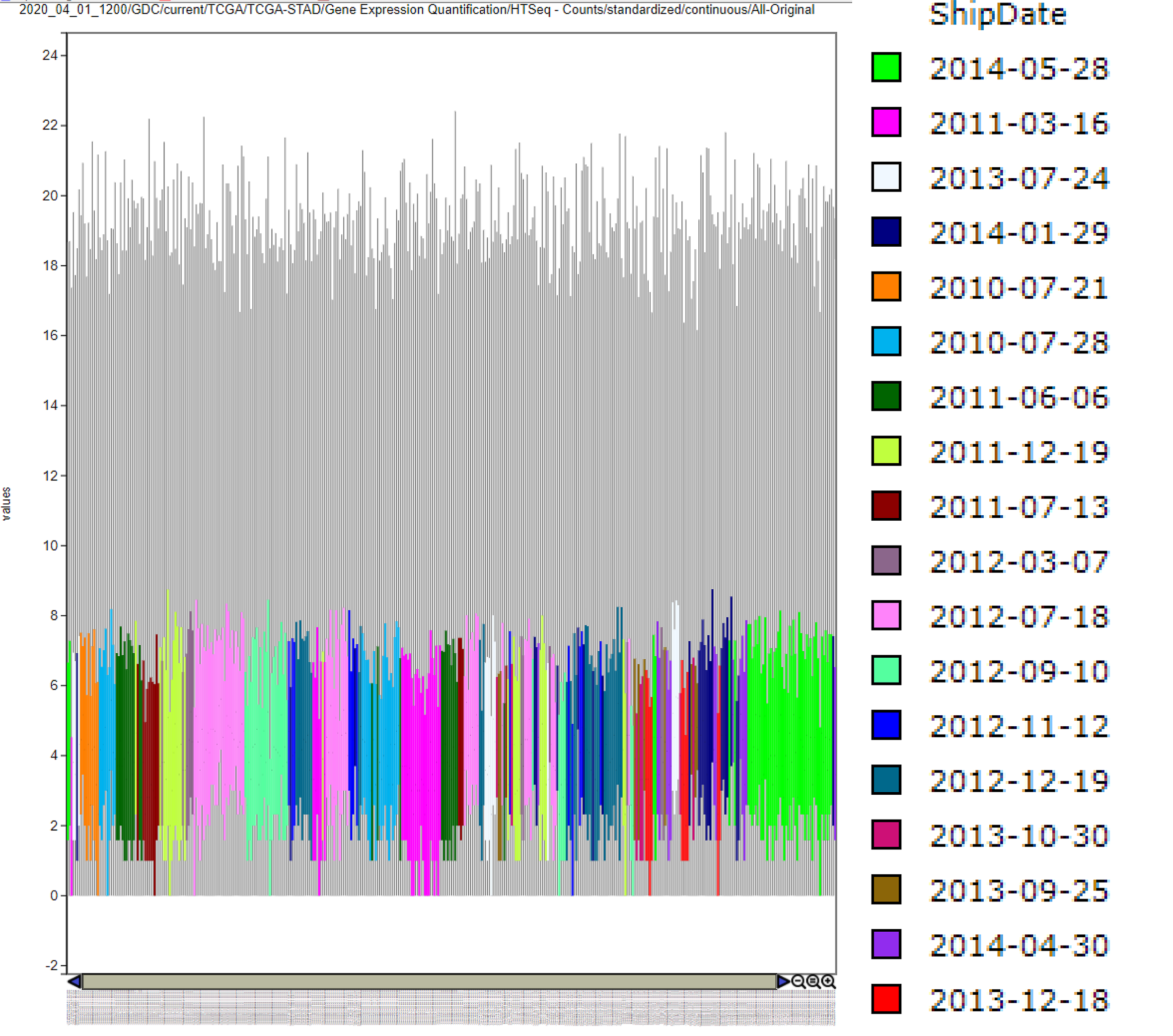

Supplement: Supplementary Figure 1 — Batches of GC TCGA data. [file Image_1.TIF]

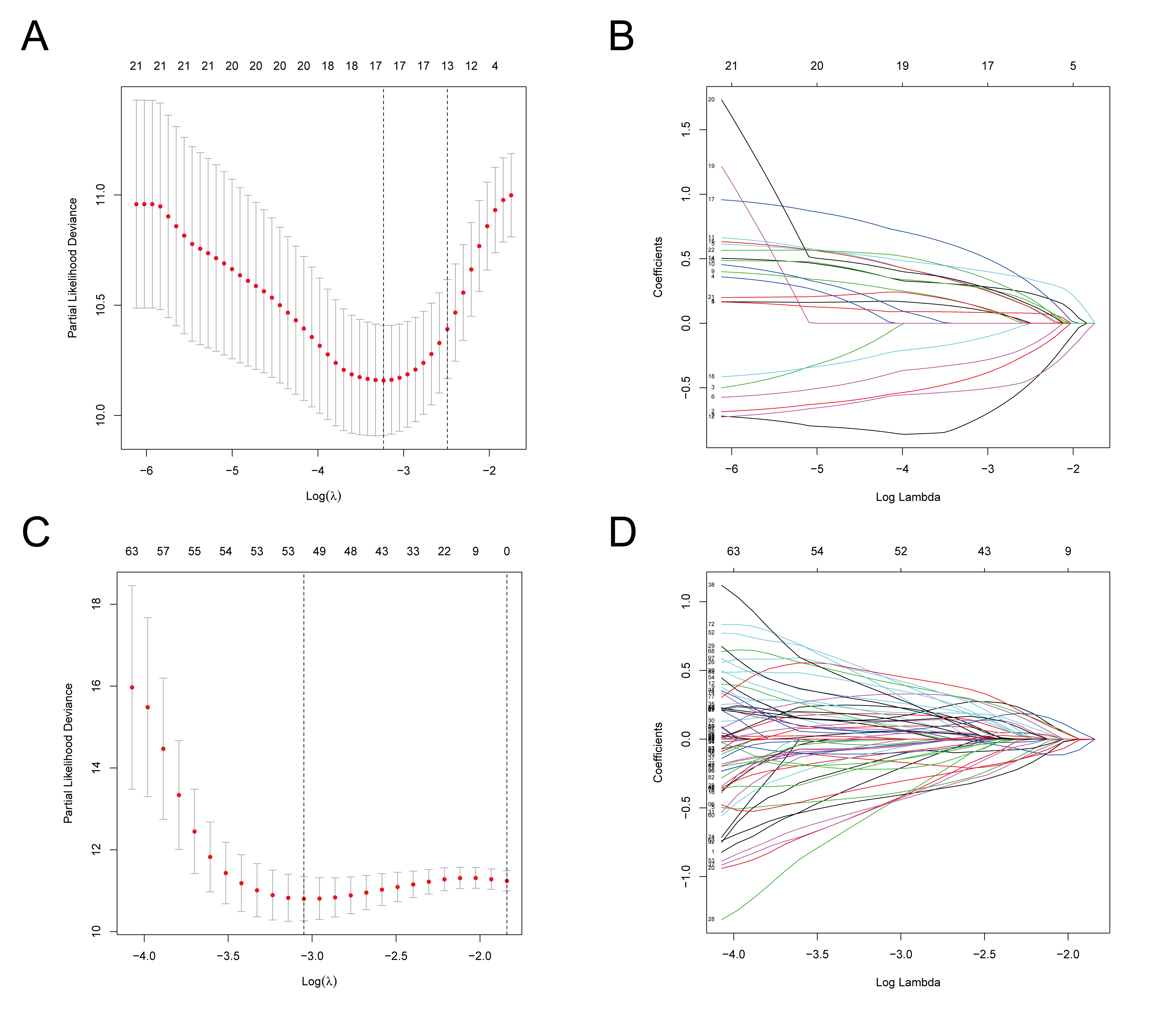

Supplement: Supplementary Figure 2 — LASSO analysis for DEAS events. (A,B) OS-related DEAS. (C,D) DFS-related DEAS. In left plot, each curve showed an OS-related DEAS event; 10-fold cross-validation was used to calculate best lambda which leads to minimum mean cross-validated error. The right plot was the partial likelihood deviance of the LASSO coefficient profiles. [file Image_2.TIF]

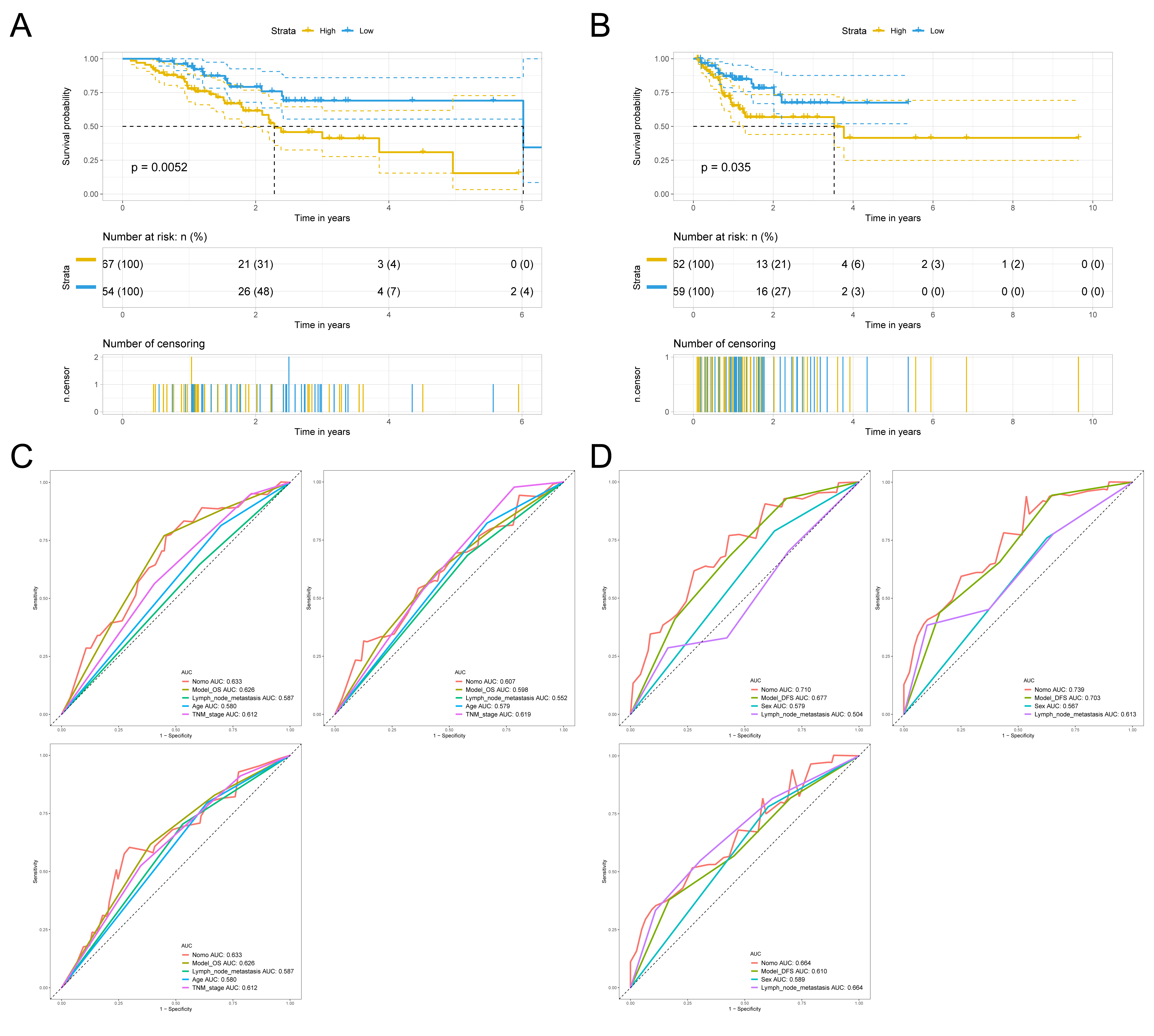

Supplement: Supplementary Figure 4 | (A,B) — KM plot of prognostic model in test group (OS and DFS). The figure contains three parts: [1] survival differences estimated by Kaplan-Meier survival curve; [2] number of patients in different groups; and [3] number censored at different times; (C,D) ROC curves with calculated AUCs of prognostic signatures built by clinical features, AS prognostic model and the nomogram for risk prediction from 1 to 3 years (OS and DFS). [file Image_4.TIF]
